# Aggravation of post-ischemic liver injury by overexpression of insulin-like growth factor binding protein 3

**DOI:** 10.1038/srep11231

**Published:** 2015-06-15

**Authors:** Lu Zhou, Hyoung-Won Koh, Ui-Jin Bae, Byung-Hyun Park

**Affiliations:** 1 Department of Sports Medicine, Taishan Medical University, Taian, Shandong, 271-000, China; 2 Department of Biochemistry, Chonbuk National University Medical School, Jeonju, Jeonbuk, 561-756, Republic of Korea

## Abstract

Insulin-like growth factor-1 (IGF-1) is known to inhibit reperfusion-induced apoptosis. IGF-binding protein-3 (IGFBP-3) is the major circulating carrier protein for IGF-1 and induces apoptosis. In this study, we determined if IGFBP-3 was important in the hepatic response to I/R. To deliver IGFBP-3, we used an adenovirus containing IGFBP-3 cDNA (AdIGFBP-3) or an IGFBP-3 mutant devoid of IGF binding affinity but retaining IGFBP-3 receptor binding ability (AdIGFBP-3^GGG^). Mice subjected to I/R injury showed typical patterns of hepatocellular damage. Protein levels of IGFBP-3 were increased after reperfusion and showed a positive correlation with the extent of liver injury. Prior injection with AdIGFBP-3 aggravated liver injury: serum aminotransferases, prothrombin time, proinflammatory cytokines, hepatocellular necrosis and apoptosis, and neutrophil infiltration were markedly increased compared to control mice. A decrease in antioxidant potential and an upregulation of NADPH oxidase might have caused these aggravating effects of IGFBP-3. Experiments using HepG2 cells and N-acetylcysteine-pretreated mice showed a discernible effect of IGFBP-3 on reactive oxygen species generation. Lastly, AdIGFBP-3 abolished the beneficial effects of ischemic preconditioning and hypothermia. Mice treated with AdIGFBP-3^GGG^ exhibited effects similar to those of AdIGFBP-3, suggesting a ligand-independent effect of IGFBP-3. Our results suggest IGFBP-3 as an aggravating factor during hepatic I/R injury.

Liver injury following ischemia reperfusion (I/R) remains a serious clinical problem affecting liver transplantation outcomes. Despite improved preservation and surgical techniques, I/R causes up to 10% of early organ failure and predisposes to chronic rejection^1^. The mechanisms underlying hepatic I/R injury are highly complex; however, it is now becoming clear that reactive oxygen species (ROS) initiate signaling cascades that lead to tissue injury^2^. ROS activate redox-regulated transcription factors activating protein-1 (AP-1) and nuclear factor-κB (NF-κB), which trigger the secretion of proinflammatory cytokines such as tumor necrosis factor-α (TNF-α), interleukin (IL)-1β, and IL-6^3^,^4^. In response to these cytokines, neutrophils are recruited into the injured area and they further release ROS, cytokines, myeloperoxidase (MPO), and various other mediators, all of which amplify tissue damage^5^. At the tissue level, ROS and cytokines activate enzymes that are involved in the processes of necrosis and apoptosis, which are types of cell death depending on the intensity and time of I/R injury^6^,^7^. On histology, this is manifested by disruption of the tissue lattice and interstitial edema.

I/R injury affects the expression of several growth factors including insulin-like growth factor-1 (IGF-1). IGF-1 has a well-established protective effect against I/R injures in liver^8^, kidney^9^, heart^10^,^11^, and brain^12^. IGF-1 is transported to its action site by binding to six structurally related IGF binding proteins (IGFBPs). Among them, IGFBP-3 is the most abundantly present and controls the actions of IGF-1 by regulating its distribution and by interacting with IGF-1 receptor^13^. Recently, several groups have provided evidence supporting intrinsic bioactivity of IGFBP-3 that is independent of IGF-1 binding^14^,^15^. IGFBP-3 sensitizes TNF-α-induced apoptosis in various cell types and increases apoptosis *in vivo* in animal models^13^,^14^,^16^. In addition, IGFBP-3 attenuates inflammation in experimental models of allergic airway inflammation^17^ and rheumatoid arthritis^16^. Given that apoptosis and inflammation are critical events for I/R injury, IGFBP-3 may play a role in the pathogenesis of I/R injury. To our knowledge, however, there are no reports on its effects against I/R injury. Because of conflicting evidence on the proapoptotic and antiinflammatory actions of IGFBP-3, we examined whether IGFBP-3 inhibited or aggravated hepatic ischemia/reperfusion (I/R) injury.

## Results

### IGFBP-3 expression is increased during hepatic I/R injury

**Figure 1:**
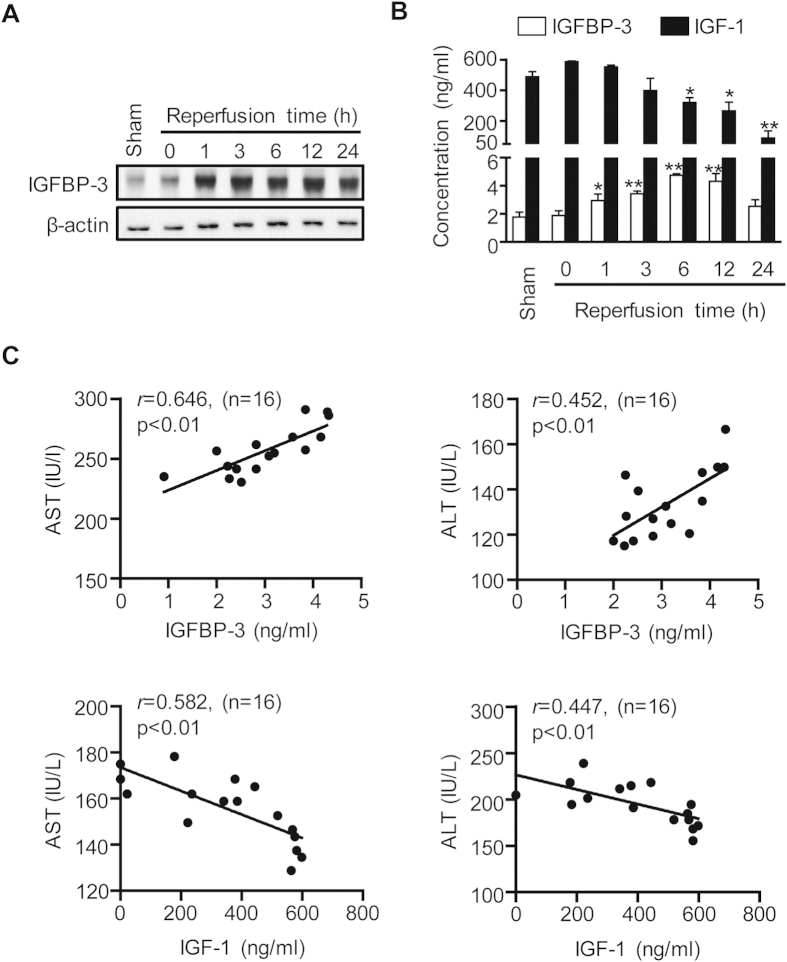
IGFBP-3 protein expression during hepatic I/R injury. Mice were subjected to 45 min of partial ischemia and liver tissues were collected after various reperfusion times. Protein levels of IGFBP-3 in liver tissue (**A)** and serum levels of IGFBP-3 and IGF-1 (**B**) were analyzed by Western blotting and ELISA, respectively. Values are the mean ± SEM (n = 9 mice per group). ^*^, *p* < 0.05 and ^**^, *p* < 0.01 versus sham. (**C**) A regression analysis was performed. The correlation coefficient (*r*), *p* value, and sample number (n) used for each analysis are shown.

To determine if IGFBP-3 plays a role in hepatic I/R injury, we first determined IGFBP-3 protein levels in reperfused liver tissues after 45 min of ischemia with various length of reperfusion. The liver protein and serum levels of IGFBP-3 remained near baseline in mice with ischemia alone, began to increase 1 h after the initiation of reperfusion, reached maximum levels at 6 h and remained elevated up to 12 h, and then declined thereafter (Figs 1A,B). To determine the correlation of IGFBP-3 production with the extent of liver injury, we compared serum IGFBP-3 levels in mice with various degrees of liver I/R injury. Mean serum levels of IGFBP-3 had a significant positive correlation with those of aspartate aminotransferase (AST) and alanine aminotransferase (ALT) (Fig. 1C). In contrast to IGFBP-3, IGF-1 levels were decreased after reperfusion (Fig. 1B) and serum levels were inversely correlated with those of aminotransferase (Fig. 1C).

Next, to assess the role of IGFBP-3 in liver I/R injury, we injected C57BL/6 mice intravenously with 1 × 10^9^ pfu of AdLacZ, AdIGFBP-3, or AdIGFBP-3^GGG^ virus (Fig. S1A). Liver tissues and blood samples were collected at various time points after adenoviral injection to analyze IGFBP-3 expression. Western blotting and ELISA analyses showed that IGFBP-3 expression peaked 2-3 days after virus injection and then declined slightly over the next few days (Figs S1B and S1C). To determine the cellular localization of IGFBP-3 expression in the liver, we performed immunohistochemistry experiments. Mice injected with AdIGFBP-3 showed immunoreactivity for IGFBP-3 in hepatocytes and endothelial cells (Fig. S1D). On the basis of the production patterns of adenovirus-mediated IGFBP-3 in the liver, I/R was performed two days after adenovirus administration (Fig. S1E). Virus injection itself did not increase aminotransferases or proinflammatory cytokines levels (data not shown).

### IGFBP-3 augments hepatic I/R injury

**Figure 2:**
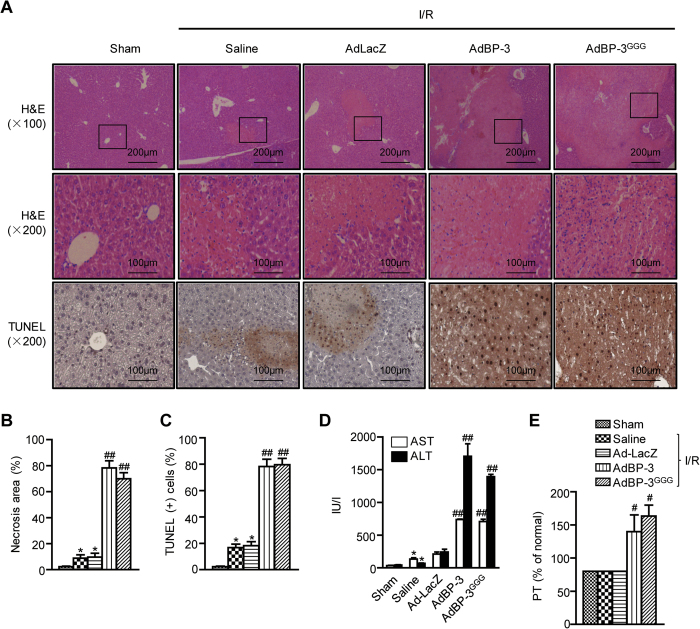
Aggravation of hepatic I/R injury by IGFBP-3. (**A**) After 24 h reperfusion, liver necrosis and apoptosis were assessed by H&E and TUNEL staining methods, respectively. (**B**) The area of necrosis was analyzed. (**C**) Apoptotic cells were counted and expressed as a percentage of all hepatocytes. (**D**) After 6 h reperfusion, serum levels of AST and ALT were analyzed. (**E**) After 24 h reperfusion, prothrombin time (PT) was analyzed. Values are the mean ± SEM (n = 9 mice per group). ^*^, *p* < 0.05 versus sham-operated mice; ^#^, *p* < 0.05 and ^##^, *p* < 0.01 versus AdLacZ-injected mice. AdBP-3, AdIGFBP-3; AdBP-3 ^GGG^, AdIGFBP-3^GGG^.

The effects of IGFBP-3 on partial hepatic I/R injury were investigated. Liver injury was assessed by histologic observation. Mice subjected to sham surgery had normal liver architecture. In saline- or AdLacZ-treated mice, typical hepatocellular necrosis was observed after 45 min of ischemia and 24 h of reperfusion (Figs 2A,B). However, these mice still retained considerable areas of normal liver architecture. Obviously, more extensive hepatocellular necrosis was observed in AdIGFBP-3-injected mice compared to those of saline- or AdLacZ-treated mice.

Although the major cause of cell death during hepatic I/R injury is necrosis, apoptotic cell death is also observed during the reperfusion process^18^. The number of apoptotic cells in I/R injured liver tissues was determined by TUNEL staining. The number of TUNEL-positive apoptotic cells was markedly increased in AdIGFBP-3-injected mice compared to that of AdLacZ-injected mice (Figs 2A,C). Consistently, increased protein levels of proapoptotic caspase-3, caspase-9, and Bax and decreased protein levels of antiapoptotic Bcl-2 were observed in IGFBP-3 overexpressing mice (Fig. S2).

These histologic findings were further confirmed by biochemical analyses of hepatocellular damage. Serum levels of ALT and AST were significantly increased after I/R injury in AdLacZ-injected mice compared to those of sham mice (Fig. 2D). Overexpression of IGFBP-3 in the liver greatly augmented the elevation of serum ALT and AST levels. In addition, prothrombin time was measured as a marker of liver synthetic function. I/R injury itself did not affect prothrombin time in sham mice 24 h after reperfusion; however, it was significantly prolonged in AdIGFBP-3-injected mice (Fig. 2E). Importantly, AdIGFBP-3^GGG^ also clearly increased hepatocellular injury to the same extent as AdIGFBP-3, suggesting that there is a ligand-independent action of IGFBP-3 on I/R-induced liver injury.

### IGFBP-3 increases neutrophil infiltration in I/R injured liver tissue

**Figure 3:**
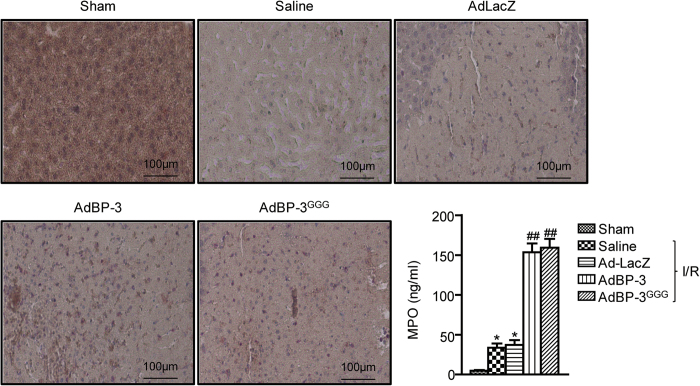
Effect of IGFBP-3 on neutrophil infiltration. Naphthol AS-D chloroacetate esterase staining was performed on liver sections after 24 h reperfusion. Neutrophils are colored red. MPO activity was determined as an index of neutrophil infiltration. Values are the mean ± SEM (n = 9 mice per group). ^*^, *p* < 0.05 versus sham-operated mice; ^##^, *p* < 0.01 versus AdLacZ-injected mice.

Accumulation of activated neutrophils in the liver plays an important role in hepatocyte death during reperfusion^5^. We used naphthol AS-D chloroacetate esterase staining, which is specific for cells of granulocytic lineage, to detect neutrophil infiltration. After 24 h of reperfusion, the number of infiltrating neutrophils was increased in the saline and AdLacZ groups, whereas the sham mice showed little neutrophil accumulation (Fig. 3). Consistent with an increase in liver damage, IGFBP-3- and IGFBP-3^GGG^-overexpressing mice both had massive neutrophil accumulation after 24 h of reperfusion.

The MPO assay, measuring MPO, an enzyme predominantly stored in azurophilic neutrophil granules, was used to quantify neutrophil infiltration in the liver. AdIGFBP-3- and AdIGFBP-3^GGG^-injected mice had almost four times as much MPO activity than that of the AdLacZ group (Fig. 3).

### IGFBP-3 increases the production of proinflammatory cytokines involved in hepatic I/R injury

**Figure 4:**
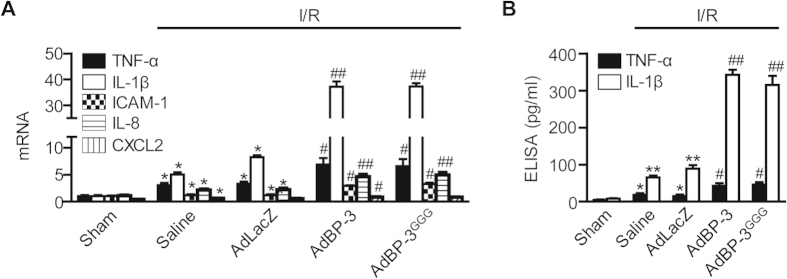
Increase of proinflammatory mediator production by IGFBP-3. (**A**) After 1 h reperfusion, mRNA levels of TNF-α, IL-1β, ICAM-1, IL-8, and CXCL2 in liver tissues were analyzed by real-time RT-PCR. (**B**) After 6 h reperfusion, serum levels of TNF-α, and IL-1β were measured using ELISA. Values are the mean±SEM (n = 9 mice per group). ^*^,  *p*< 0.05 and ^**^, *p* < 0.01 versus sham-operated mice; ^#^, *p* < 0.05 and ^##^, *p* < 0.01 versus AdLacZ-injected mice.

Proinflammatory cytokines are upregulated by hepatic I/R and are responsible for the pathophysiological changes in I/R injury^19^. To further examine the role of cytokines in hepatic I/R injury, mRNA levels of IL-1β, TNF-α, intercellular adhesion molecule-1 (ICAM-1), IL-8, and chemokine ligand 2 (CXCL2) were measured by real-time RT-PCR. We observed a significant increase in mRNA levels of the aforementioned cytokines after hepatic I/R injury (Fig. 4A). Prior injection with AdIGFBP-3 or AdIGFBP-3^GGG^ resulted in a marked increase of IL-1β, moderate increase of TNF-α, and mild increases of ICAM-1, IL-8, and CXCL2. In addition, changes in serum TNF-α and IL-1β levels were similar to those seen for mRNA expression (Fig. 4B).

### IGFBP-3 eliminates the protective effects of ischemic preconditioning and hypothermia

**Figure 5:**
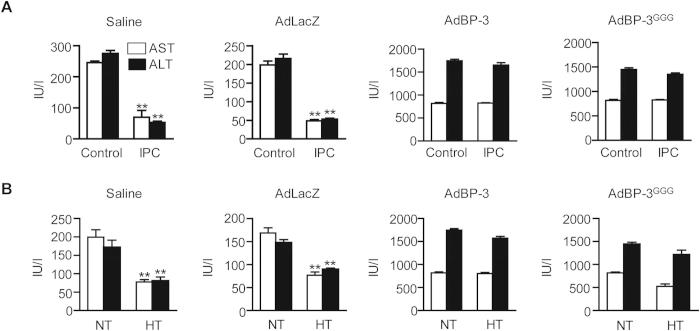
Elimination of the protective effect of ischemic preconditioning and hypothermia by IGFBP-3. (**A**) Before I/R injury, mice were subjected to 10 min of ischemia and 10 min reperfusion (IPC). Control mice were subjected to I/R without IPC. Serum levels of AST and ALT were measured. (**B**) Mice underwent 45 min of ischemia and 6 h reperfusion at normothermia (37 °C) or moderate hypothermia (32 °C) and serum levels of AST and ALT were measured. Values are the mean ± SEM (n = 12 mice per group). ^**^, *p* < 0.01 versus sham- or NT-operated mice. NT, normothermia; HT, hypothermia.

Ischemic preconditioning (IPC), a brief period of I/R before sustained ischemia, is an endogenous protective mechanism that makes the liver more tolerant to subsequent prolonged ischemia^20^. To evaluate the effects of IGFBP-3 on hepatic IPC, mice were first subjected to 10 min of ischemia followed by 10 min of reperfusion and finally by 45 min of ischemia. The control mice were subjected to 45 min of ischemia without preconditioning. Interestingly, IPC itself significantly decreased the plasma concentration of IGFBP-3 (Fig. S3A). In saline- or AdLacZ virus-treated mice, IPC significantly reduced the elevation of serum AST and ALT levels after 6 h of reperfusion compared to mice that had not been preconditioned (Fig. 5A). However, in AdIGFBP-3 and AdIGFBP-3^GGG^-treated mice, serum AST and ALT levels were increased to levels similar to those of sham mice.

Whole body moderate hypothermia (32–34 °C) during I/R injury or preservation of liver grafts at 4 °C before transplantation prolongs tolerance against I/R and is a powerful strategy for limiting hepatic I/R injury^21^. When the mice were kept at 4 °C for 2 days, a significant decrease in IGFBP-3 was observed (Fig. S3B). Similar to IPC, I/R injury at hypothermic conditions was attenuated in saline- or AdLacZ-treated mice, and the beneficial effects of hypothermia were abolished in AdIGFBP-3- and AdIGFBP-3^GGG^-injected mice (Fig. 5B).

### IGFBP-3 potentiates oxidative stress

**Figure 6:**
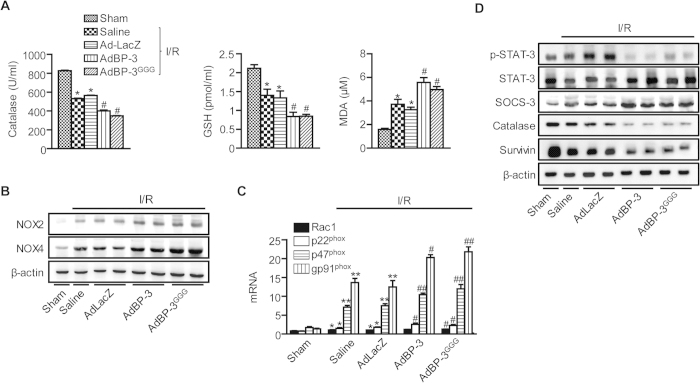
Effect of IGFBP-3 overexpression on I/R-induced oxidative stress. (**A**) After 6 h reperfusion, tissue levels of catalase, GSH, and MDA were analyzed. (**B**) After 24 h reperfusion, NOX2 and NOX4 levels were examined by Western blotting. (**C**) After 1 h reperfusion, mRNA levels of Rac1, p22^phox^, p47^phox^, and gp91^phox^ in liver tissues were analyzed by real-time RT-PCR. (**D**) After 24 h reperfusion, and p-STAT-3, SOCS-3, catalase, and survivin levels were examined by Western blotting. Values are the mean ± SEM (n = 12 mice per group). ^*^, *p* < 0.05 and ^**^, *p* < 0.01 versus sham-operated mice; ^#^, *p* < 0.05 and ^##^, *p* < 0.01 versus AdLacZ-injected mice. MDA, malondialdehyde; GSH, glutathione.

I/R injury is partially mediated by oxidative stress^2^, and IGFBP-3 is known to increase oxidative stress in *in vitro* cell culture conditions^22^. Therefore, we hypothesized that IGFBP-3 may play a role in hepatic I/R injury in mice by regulating oxidative stress. I/R caused significant suppression of hepatic antioxidant potential, as observed by decreases in catalase enzyme activities and glutathione levels (Fig. 6A). In contrast, serum levels of malondialdehyde (MDA), an indicator of oxidative damage, were significantly increased by I/R. IGFBP-3 potentiated these changes caused by I/R; hepatic levels of catalase and glutathione were significantly lower, and serum levels of MDA were significantly higher in IGFBP-3-overexpressing mice than in I/R mice with saline or AdLacZ treatment. Because we observed increased neutrophil infiltration by IGFBP-3 in I/R injured liver tissues, we analyzed activation of NADPH oxidase (NOX). Compared to AdLacZ, IGFBP-3 overexpression markedly increased the protein levels of NOX-2 and NOX-4 as well as mRNA levels of NOX components such as Rac1, p22^phox^, p47^phox^, and gp91^phox^ (Fig. 6C). During hepatic I/R injury, hypophosphorylation of signal transducer and activator of transcription 3 (STAT-3) leads to increases in ROS production and apoptosis^23^. We therefore compared the STAT-3 signaling pathway in treated and control groups. Compared to AdLacZ group, IGFBP-3 overexpression led to decreases in levels of p-STAT-3, catalase, and survivin as well as increase in levels of suppressor of cytokine signaling 3 (SOCS-3) (Fig. 6D).

### IGFBP-3 increases apoptosis after H/R injury in HepG2 cells

To further validate the aggravating activity of IGFBP-3 in I/R-injured liver tissue, we performed *in vitro* studies using a hypoxia-reoxygenation (H/R) model. HepG2 cells were cultured in anaerobic jars for 24 h and reoxygenated for 6 h, at which time they were harvested and IGFBP-3 expression levels were compared. Similar to the results observed in I/R-injured liver tissues, H/R markedly increased protein levels of IGFBP-3 (Fig. S4A). ROS production, determined by DCF-DA fluorescence, was significantly increased by IGFBP-3 overexpression (Fig. S4B), whereas the expression levels of catalase were significantly decreased (Fig. S4C) compared to control cells. In the presence of N-acetlycysteine (NAC), these changes were almost completely inhibited.

We further assayed the apoptotic effects of IGFBP-3 using an ApoPercentage Apoptosis staining kit. As shown in Fig. S5A, more apoptotic cells were observed in AdIGFBP-3-treated cells. Annexin V staining also showed a significant increase of apoptotic cells in AdIGFBP-3-treated cells compared with AdLacZ-treated cells (7.11 ± 1.45% versus 20.25 ± 5.05%,  *p* < 0.01) (Fig. S5B). Consistently, increased protein levels of caspase-3 and Bax and decreased protein levels of Bcl-2 were observed in AdIGFBP-3 treated cells (Fig. S5C). Again, NAC attenuated the effects of IGFBP-3 overexpression on apoptosis.

### NAC abrogates IGFBP-3-mediated aggravation of hepatic I/R injury

**Figure 7:**
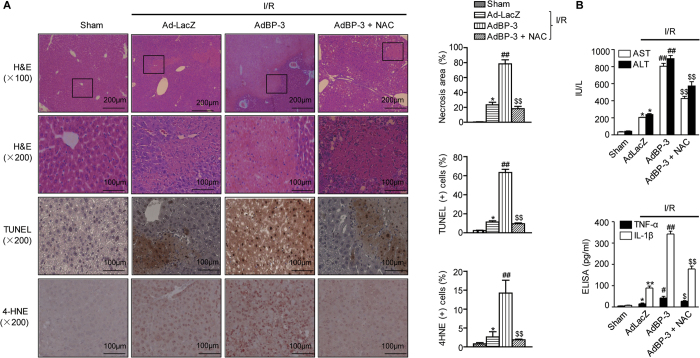
Abrogation of IGFBP-3-induced aggravation of hepatic I/R injury by NAC pretreatment. (**A**) Mice were pretreated with 10 μM NAC *via* intravenous injection before I/R injury. After 24 h reperfusion, liver tissues were stained with H&E, TUNEL, and 4-HNE. The area of necrosis and the percentages of apoptotic and 4-HNE positive cells were analyzed. (**B**) Serum aminotransferase and cytokine levels were analyzed. Values are the mean ± SEM (n = 9–12 mice per group). ^*^, *p* < 0.05 and ^**^, *p* < 0.01 versus sham-operated mice; ^#^, *p* < 0.05 and ^##^, *p* < 0.01 versus AdLacZ-injected mice; ^$^, *p* < 0.05 and ^$$^, *p* < 0.01 versus Ad-BP-3-injected mice.

Because NAC effectively abolished H/R-induced cell damage, we pretreated mice with NAC before I/R injury. NAC pretreatment effectively abrogated the aggravating effects of IGFBP-3, as indicated by reduced parenchymal necrosis and apoptosis and suppressed ROS production (Fig. 7A). Levels of serum aminotransferase and cytokines were also significantly downregulated by NAC (Fig. 7B).

## Discussion

According to previous reports, IGFBP-3 might aggravate hepatic I/R injury because IGFBP-3 induces apoptosis by both IGF-dependent and IGF-independent mechanisms^13^ and because IGFBP-3 produces ROS^22^. In contrast, IGFBP-3 could protect hepatic I/R injury because IGFBP-3 inhibits NF-κB activity and suppresses inflammatory responses^13^,^16^,^17^. Therefore, the aim of the present study was to investigate the response of IGFBP-3-overexpressing mice against I/R injury. The results revealed that IGFBP-3 worsened morphometric tissue damage, aminotransferase release, and inflammatory responses with concomitant changes in biochemical parameters following I/R. Of interest, IGF-1 administration protects several organs against I/R injury and improves functional recovery^8^,^9^,^10^,^11^,^12^. To exclude the possible involvement of IGF-l, we used an IGFBP-3 mutant with disrupted binding affinity for IGF-1 due to mutations in the binding site. Similar to IGFBP-3, mutant IGFBP-3 also exacerbated liver damage. These results suggest that the aggravating effects of IGFBP-3 on tissue damage during hepatic I/R were independent on IGF-1 binding.

During I/R injury, we observed increases in hepatic and serum levels of IGFBP-3 as well as a decrease in serum levels of IGF-1. In addition, serum levels of IGFBP-3 were positively correlated with serum levels of aminotransferase, while IGF-1 had an inverse correlation. These results are not consistent with Casillas-Ramírez A *et al.*^24^, in which I/R led to hepatic and circulating levels of IGF-1 and IGFBP-3 similar to those of the sham group. This discrepancy may result from the differences in the experimental protocols. In our protocol, mice were subjected 45 min ischemia and various lengths of reperfusion, whereas Casillas-Ramírez A *et al.* used 60 min ischemia and 24 h reperfusion. In addition, differences in animal species (C57BL/6 mice versus Zucker rats) can contribute to the different outcomes. Using C57BL/6 mice, we observed that the serum levels of IGFBP-3 began to increase after reperfusion, reached maximum levels at 6 h, and returned to the sham group levels at 24 h reperfusion. Casillas-Ramírez A *et al* used Zucker rats and found similar levels of IGF-1 and IGFBP-3 in lean Zucker rats compared with sham group. When obese Zucker rats were used, after 24 h reperfusion, similar results were observed between the studies. Of note, we further observed suppressed serum levels of IGFBP-3 during IPC and hypothermia. Given that IPC and hypothermia have stood out as leading therapeutic approaches to protect against I/R injury over past two decades^20^,^21^, an increase of IGFBP-3 during hepatic I/R injury could lead to the deleterious outcome in hepatic I/R injury.

ROS is known to initiate apoptosis and subsequent inflammatory cell infiltration including neutrophils^2^. Thus, limiting oxidative stress during I/R has been a very effective way to reduce tissue injury during I/R. For example, several antioxidants such as hydroxytyrosol^25^, vitamin E^26^, and α-lipoic acid^27^ are effective in protecting against tissue injury and inflammatory responses in I/R-injured liver tissue. In addition, ischemic preconditioning^2^,^28^ and hypothermia^29^,^30^ result in less parenchymal apoptosis during I/R injury through antioxidant mechanisms. In contrast, increased hepatic ROS production by disruption of Notch signaling aggravates hepatic I/R injury by attenuating STAT-3 phosphorylation and down-regulating MnSOD^23^. Previously, Yoo *et al.*^22^ reported that IGFBP-3 overexpression in proximal tubular epithelial cells increases ROS production and induces high glucose-induced apoptosis. Consistent with these reports, our *in vivo* results showed that IGFBP-3 overexpression depleted hepatic glutathione (GSH) content, reduced enzyme activity of catalase, and increased hepatic MDA level, indicating that IGFBP-3 could effectively augment I/R-induced injury by increasing oxidative stress in the liver.

At present, sources of ROS production by IGFBP-3 during I/R injury are not clear. During I/R, ROS are produced from the mitochondria when hepatocytes are exposed to cytokines^31^. ROS can also be produced from Kupffer cells, infiltrating neutrophils, and endothelial cells^6^,^7^. Because we observed a marked infiltration of neutrophils by IGFBP-3, we thought it is likely that neutrophilic NOX might be the source of ROS production. Specifically, we focused on the most relevant isoforms, NOX2 and NOX4, whose roles during the hepatic I/R injury have already been implicated^32^,^33^. Results showed that IGFBP-3 increased the expression of NOX2 and NOX4 as well as several NOX components. Because IGFBP-3 further decreased catalase activity and increased MPO activity, it is thus possible that superoxide anion produced by neutrophilic NOX is converted to H_2_O_2_, which provides ample substrate for MPO to form hypochlorous acid (HOCl). While the production of HOCl may be injurious by inactivating sulfhydryl-containing proteins^34^, it is going to further result in a decrease of GSH content and an increase of MDA level in the liver tissues. In addition, NOX-mediated ROS production has a major role in the I/R-induced inflammatory responses: hepatocytes produce TNF-α in a NOX-dependent manner following H/R or I/R injury^35^. On the other hand, proinflammatory cytokines are involved in the ROS production^2^,^5^. These bidirectional relationships interact and amplify each other, finally leading to functional and structural liver damage.

The mechanism by which IGFBP-3 increases ROS production may involve, at least in part, decreased STAT-3 activation, which further attenuates antioxidant enzyme expression^23^. Because SOCS-3 prevents cytokine signaling by inhibiting Janus kinase activity or by preventing STAT recruitment to the receptor, decreased STAT-3 activation seems to be associated with increased expression of SOCS-3. Further studies are required to clarify the exact mechanism for STAT-3 hypophosphorylation by IGFBP-3.

New marker(s) of hepatic I/R injury may provide mechanistic insights and reveal therapeutic possibilities. Here, we propose IGFBP-3 as an aggravating factor during hepatic I/R injury. We provide evidence supporting this conclusion. First, we observed that expression of IGFBP-3 in liver tissue and its release into systemic circulation were increased during I/R injury, and serum IGFBP-3 levels were closely correlated with the degree of liver injury. Second, mice exposed to IPC or hypothermia before I/R injury had lower serum levels of IGFBP-3. Third, adenoviral-mediated overexpression of IGFBP-3 resulted in more tissue injury following I/R. Fourth, IGFBP-3 increases ROS production, cytokines release, and apoptosis. Our *in vitro* studies provide additional evidence supporting this concept.

## Materials and Methods

### Cell culture and reagents

HepG2 cells were obtained from the American Type Culture Collection (Manassas, VA, USA) and maintained in Dulbecco’s Modified Eagle Medium (DMEM) supplemented with 10% fetal bovine serum.

### Preparation of recombinant adenovirus

Adenoviruses containing IGFBP-3 and IGFBP-3^GGG^ were kindly donated by Y. Oh (Virginia Commonwealth University, Richmond, VA, USA). HEK293 cells were used for viral transfection and amplification. Viruses from the culture supernatants of HEK 293A cells with cytopathogenic effects were purified by cesium chloride banding. Virus (1 × 10^9^ pfu) was intravenously administrated to mice before I/R operation.

### Hypoxia-reoxygenation protocol

HepG2 cells in DMEM were incubated at 37 °C in anaerobic jars (Oxoid, Basingstoke, Hampshire, England) with oxygen absorbing packs (AnaeroGen, Oxoid). This method has been shown to achieve oxygen levels in the jar to below 1%. Following 24 h of hypoxia, reoxygenation of hepatocytes was started by opening the chamber and replacing the hypoxic medium with oxygenated medium. Cells were exposed to 1 × 10^6^ pfu of AdLacZ, AdIGFBP-3, or AdIGFBP-3^GGG^ for 24 h prior to placing them in an anaerobic jar.

### Assessment of ROS production

Generation of ROS was measured using 2′,7′-dichlorodihydrofluorescein diacetate (DCF-DA).

### Annexin V staining

Cells were cultured in aerobic jars for 24 h. Cells were stained with Annexin V according to the manufacturer’s instructions (Invitrogen, Carlsbad, CA, USA). Percentages of apoptotic cells were analyzed by flow cytometry performed on a BD Accuri flow cytometer (BD Biosciences, San Jose, CA, USA).

### Animals

Pathogen-free 8–10-week-old C57BL/6 male mice (Orient, Seoul, Korea) were maintained on standard laboratory chow and water *ad libitum*. All animal experiments were performed in accordance with the Guide for the Care and Use of Laboratory Animals, published by the US National Institutes of Health (NIH Publication No. 85-23, revised 2011). The current study protocol was also approved by the Institutional Animal Care and Use Committee of Chonbuk National University (Approval No. CBU 2014-1-0221).

### Model of partial hepatic I/R injury

Hepatic ischemia was created by occluding portal vein, hepatic artery, and bile duct just above the right branch, which represent approximately 70% of the total blood supply to the liver as described previously^4^. For ischemic preconditioning experiments, mice were subjected to 10 min of partial hepatic ischemia following 10 min of reperfusion prior to 45 min of ischemia. For whole-body moderate hypothermic experiments, mice were exposed to ambient room temperature and passively cooled to achieve a rectal temperature of 32 °C.

### Quantification of liver injury and cytokines

Serum ALT and AST levels were measured using a commercial kit from Asan Pharm (Seoul, Korea). Hepatocellular function was evaluated by measuring prothrombin time, which was quantified using a portable coagulometer (CoaguChek XS^®^, Roche Diagnostics, Mannheim, Germany). Serum levels of IGFBP-3, TNF-α, IL-1β (Invitrogen), and IGF-1 (R&D Systems, Minneapolis, MN, USA) were measured using ELISA.

### Western blot analysis

Liver homogenates containing 10 μg of whole cell lysate were separated by 10% SDS-PAGE and transferred to PVDF membranes. After blocking with 5% skim milk, the blot was probed with primary antibodies for IGFBP-3, survivin, SOCS-3, NOX4, β-actin (Santa Cruz Biotechnology, Dallas, TX, USA), Bax, Bcl-2, cleaved caspase-3, cleaved caspase-9, STAT-3, p-STAT-3, catalase (Cell Signaling Technology, Beverly, MA, USA), and NOX2 (Abcam, Cambridge Science Park, Cambridge, UK).

### Histological study

Fixed liver tissues were embedded in paraffin. Tissue sections (5 μm) were stained with hematoxylin and eosin (H&E) for light microscopy. TUNEL staining was performed with commercial kits (Promega, Madison, WI, USA). Five to six random sections were investigated per slide to determine the necrotic area and percentage of apoptotic cells. To measure the necrotic area, sections were observed under an Axiovert 40 CFL microscope (Carl Zeiss, Oberkochen, Germany) and measured using iSolution DT 36 software (Carl Zeiss). For immunohistochemistry, sections were immunostained with antibody against IGFBP-3 (Santa Cruz Biotechnology) or 4-hydroxynonenal (4-HNE, Abcam).

### Liver neutrophil accumulation

A naphthol AS-D chloroacetate esterase kit (Sigma-Aldrich, St. Louis, MO, USA) was used for neutrophil esterase staining of liver sections. Liver MPO activity was analyzed as a measure of neutrophil accumulation^36^.

### RNA isolation and real-time RT-PCR

Total RNA was extracted from frozen liver tissue using Trizol reagent (Invitrogen). First-strand cDNA was generated using the random hexamer primer provided in the first-strand cDNA synthesis kit (Applied Biosystems, Foster City, CA, USA). Specific primers for each gene (Supplementary Table 1) were designed using primer express software (Applied Biosystems).

### Activities of catalase, GSH, and MDA

The enzyme activity of catalase, and the levels of GSH and MDA in liver tissues were determined using commercial assay kits (Enzo Life Sciences, Plymouth Meeting, PA, USA).

### Statistical analysis

Data are expressed as means ± SEM. Statistical analyses were performed using one-way ANOVA and Duncan’s tests through GraphPad Prism version 5.02. Differences with a *p* < 0.05 were considered statistically significant.

### Additional methods

Detailed methods are provided in the Supplementary Methods.

## Additional Information

**How to cite this article**: Zhou, L. *et al.* Aggravation of post-ischemic liver injury by overexpression of insulin-like growth factor binding protein 3. *Sci. Rep.* **5**, 11231; doi: 10.1038/srep11231 (2015).

## Supplementary Material

Supplementary Information
