# Supplementary material for: Aggravation of post-ischemic liver injury by overexpression of insulin-like growth factor binding protein 3
Source: Sci Rep. 2015 Jun 15;5:11231. doi: 10.1038/srep11231 (PMC4466889; doi:10.1038/srep11231)
Supplement: Supplementary Information [file srep11231-s1.pdf]

Aggravation of post-ischemic liver injury by overexpression of insulin-like growth factor binding protein 3

Lu Zhou, PhD[a,b], Hyoung-Won Koh[b], Ui-Jin Bae[b], and Byung-Hyun Park, MD, PhD[b]

[a]Department of Sports Medicine, Taishan Medical University, Taishan, Shandong, 271-000, China

[b]Department of Biochemistry, Chonbuk National University Medical School, Jeonju, Jeonbuk, 561-756, Republic of Korea

Contents

1. Supplementary Methods
2. Supplementary Table
3. Supplementary Figure Legends
4. Supplementary Figures

1. Supplementary Methods

Adenovirus preparation

Adenovirus expressing IGFBP-3 (AdIGFBP-3) or IGFBP-3 mutant with a lack of IGF binding affinity (AdIGFBP-3^{GGG}) under the control of a cytomegalovirus promoter was donated by Y. Oh (Virginia Commonwealth University, Richmond, VA, USA). The control recombinant replication-defective adenovirus containing LacZ (Ad-LacZ) were obtained from R.H. Unger (University of Texas Southwestern Medical Center, Dallas, TX, USA). Viruses were purified from the supernatants of 293 cells cultures by cesium chloride density gradient centrifugation. Virus titers were determined by a plaque assay using serial dilution. Virus (1×10^9 pfu) was intravenously administrated to mice before I/R operation, and AdIGFBP-3 or AdIGFBP-3^{GGG} protein levels were measured using Western blotting and ELSIA.

Liver neutrophil accumulation

A naphthol AS-D chloroacetate esterase kit (Sigma-Aldrich) was used for neutrophil esterase staining of liver sections. Liver myeloperoxidase (MPO) activity was analyzed as a measure of neutrophil accumulation (R&D Systems). Briefly, liver samples were homogenized in PBS and centrifuged for 20 min at 13,000 *g* at 4°C. The pellet was resuspended in 1% hexadecyltrimethylammonium in 50 mM KH_2PO_4, 5 mM EDTA, pH 6.0 and sonicated for 10 sec. After heating for 2 h at 60°C, samples were centrifuged for 15 min at 13,000 *g* and the supernatant was reacted with 3, 3′, 3, 5′-tetramethylbenzidine following the addition of H_2O_2. The change of optical density was read at 650 nm.

RNA isolation and real-time RT-PCR

Total RNA was extracted from frozen liver tissue using Trizol reagent (Invitrogen). RNA was precipitated with isopropanol and dissolved in diethylpyrocarbonate-treated distilled water. Total RNA (2 μg) was treated with RNase-free DNase (Invitrogen), and first-strand cDNA was generated using the random hexamer primer provided in the first-strand cDNA synthesis kit (Applied Biosystems). Specific primers for each gene (Supplementary Table 1) were designed using primer express software (Applied Biosystems). Glyceraldehyde-3-phosphate dehydrogenase (GAPDH) was used as an invariant control. The real-time RT-PCR reaction mixture consisted of 10 ng reverse transcribed RNA, 200 nM forward and reverse primers, and 2 × PCR master mixture in a final volume of 10 μl. The PCR reaction was carried out in 384-well plates using the ABI Prism 7900HT Sequence Detection System (Applied Biosystems).

Annexin V staining

HePG2 cells were seeded in 6 cm plates and incubated at 37℃ in anaerobic jars for 24 h. Treated cells were washed twice with cold PBS and resuspended in buffer at a concentration of 10^6 cells per ml. Cells were mixed with 10 μl of fluorescein isothiocyanate (FITC)-conjugated Annexin-V reagent and 10 μl of 3 mM propidium iodide (PI). After 15 min incubation at room temperature in the dark and further washings, samples were analyzed by flow cytometry. Flow cytometry was performed with a FACScan analyzer (BD Biosciences, San Jose, CA, USA) with a 15 mW argon ion laser (488 nm) and Cell Quest software. Annexin-V staining was detected in the FL1 channel, whereas PI staining was monitored in the FL2 channel: appropriate quadrants were set and the percentage of cells negative for stains (viable cells), positive for Annexin-V (apoptotic cells), and positive for PI (dead cells)

were acquired.

Assessment of reactive oxygen species (ROS) production

HepG2 cells (1×10^5 cells/well) were incubated at 37℃ in anaerobic jars for 24 h and then reoxygenated for 6 h. After incubating cells with 10 μM DCF-DA for 45 min at 37℃, all subsequent steps were performed in the dark. Cells were then washed in PBS, harvested, and resuspended in 500 µl of Binding Buffer (BD Biosciences). Cells were then analyzed by flow cytometry using a GFP signal detector with excitation wavelength of 488 nm and emission of 530 nm (to detect DCF-DA) and a PE Texas Red signal detector with excitation wavelength of 496 nm and emission of 615 and (to detect RFP-positive cells).

2. Supplementary Table

Table S1. Sequences and accession numbers for primers (FOR, forward and REV, reverse) used in real-time RT-PCR.

Gene	Sequences for primers	Accession No.
TNF-α	FOR: AGGGTCTGGGCCATAGAACT<br>REV: CCACCACGCTCTTCTGTCTAC	NM_013693
IL-1β	FOR: GGTCAAAGGTTTGGAAGCAG<br>REV: TGTGAAATGCCACCTTTTGA	NM_008361
ICAM-1	FOR: AACAGTTCACCTGCACGGAC<br>REV: GTCACCGTTGTGATCCCTG	NM_010493
Rac1	FOR: CGTCCCGTAGACAAAATGGT<br>REV: TTGATGGCAACAATCTCCAC	NM_009007
$gp91^{phox}$	FOR: TCTCCAGGAAATGCATTGGT<br>REV: AGATGCAGGCCATCAAGTGT	NM_007807
$p47^{phox}$	FOR: GGTGATGACCACCTTTTGCT<br>REV: ACTGCGGAGAGTTTGGAAGA	NM_010876
$p22^{phox}$	FOR: CTGTTCCCGAACTCTTCTCG<br>REV: ATACTTCAACGGCCTCATGG	NM_007806
CXCL2	FOR: ACCGACAACAGGAAGTGGAG<br>REV: TGGACGTTTCACACAGTGGT	NM_009140
IL-8	FOR: TCCAGGTCAGTTAGCCTTGC<br>REV: CGGTCAAAAAGTTTGCCTTG	NM_011339
GAPDH	FOR: CGTCCCGTAGACAAAATGGT<br>REV: TTGATGGCAACAATCTCCAC	NM_008084

3. Supplementary Figure Legends

Figure S1. IGFBP-3 expression after adenovirus injection and a schematic diagram of the experimental protocol. (A) Mice were intravenously injected with 1×10^9 pfu of AdLacZ, AdIGFBP-3, or AdIGFBP-3^{GGG}. Liver tissue homogenates were prepared two days after the virus injection and the expression levels of IGFBP-3 were compared among groups. (B) After adenoviral injection, liver homogenates prepared at the indicated time points were subjected to Western blotting. (C) IGFBP-3 release into blood was determined by ELISA. Values are the mean±SEM (n=9 mice per group). **, $p<0.01$ versus day 0. (D) Two days after adenovirus injection, liver tissues were sectioned and immunostained with an IGFBP-3 antibody (×200). Arrowheads and arrows indicate IGFBP-3-expressing hepatocytes and endothelial cells, respectively. (E) Mice were intravenously injected with viruses 2 days before surgery. Blood samples and liver tissues were collected and the following analyses were performed after the reperfusion: real-time RT-PCR analysis (RT-PCR) for proinflammatory mediators (1 h); aspartate aminotransferase (AST) and alanine aminotransferase (ALT), ELISA for cytokines (6 h); myeloperoxidase (MPO), Western blotting (WB) for apoptotic proteins, prothrombin time (PT), malondialdehyde, catalase, NADPH oxidase, glutathione, and end-point histology (24 h).

Figure S2. Effect of IGFBP-3 on I/R-induced apoptosis. Liver tissues were retrieved 24 h after reperfusion and the expression levels of cleaved caspase-9, cleaved caspase-3, Bax, and Bcl-2 were examined by Western blotting.

Figure S3. Decrease of IGFBP-3 in mice exposed to ischemic preconditioning and

hypothermia. Mice were subjected to with or without ischemic preconditioning (A) or kept at room temperature or 4°C for 2 days (B). Serum levels of IGFBP-3 were analyzed by ELISA. Values are the mean±SEM (n=12 mice per group). **, *p*<0.01 versus sham or normothermic mice. NT, normothermia; HT, hypothermia; IPC, ischemic preconditioning.

Figure S4. Increase of ROS production by IGFBP-3. (A) HepG2 cells (1×10^5 cells/well) transfected with AdLacZ, AdIGFBP-3, or AdIGFBP-3^{GGG} were cultured in anaerobic jars for 24 h and reoxygenated for 6 h. Protein levels of IGFBP-3 were analyzed by Western blotting. (B) HepG2 cells (1×10^5 cells/well) preloaded with a fluorogenic probe (CM-H_2DCFDA) were subjected to H/R injury in the presence or absence of 10 μM N-acetylcysteine (NAC), and the fluorescence change of the oxidized probe was determined. (C) Catalase activity was analyzed. Values are the mean±SEM (n=4 mice per group). *, *p*<0.05 versus AdLacZ-treated cells; $^{\#}$, *p*<0.05 versus vehicle (VEH)-treated cells.

Figure S5. Aggravation of H/R injury by IGFBP-3. All experimental procedures are the same as those described in the Figure S4 legend. Apoptosis was determined by ApoPercentage Apoptosis staining (A), Annexin V staining (B), and Western blotting (C). Values are the mean±SEM (n=4 mice per group). *, *p*<0.05 versus AdLacZ-treated cells; $^{\#}$, *p*<0.05 versus vehicle (VEH)-treated cells.

Supplementary Figures

Figure S1

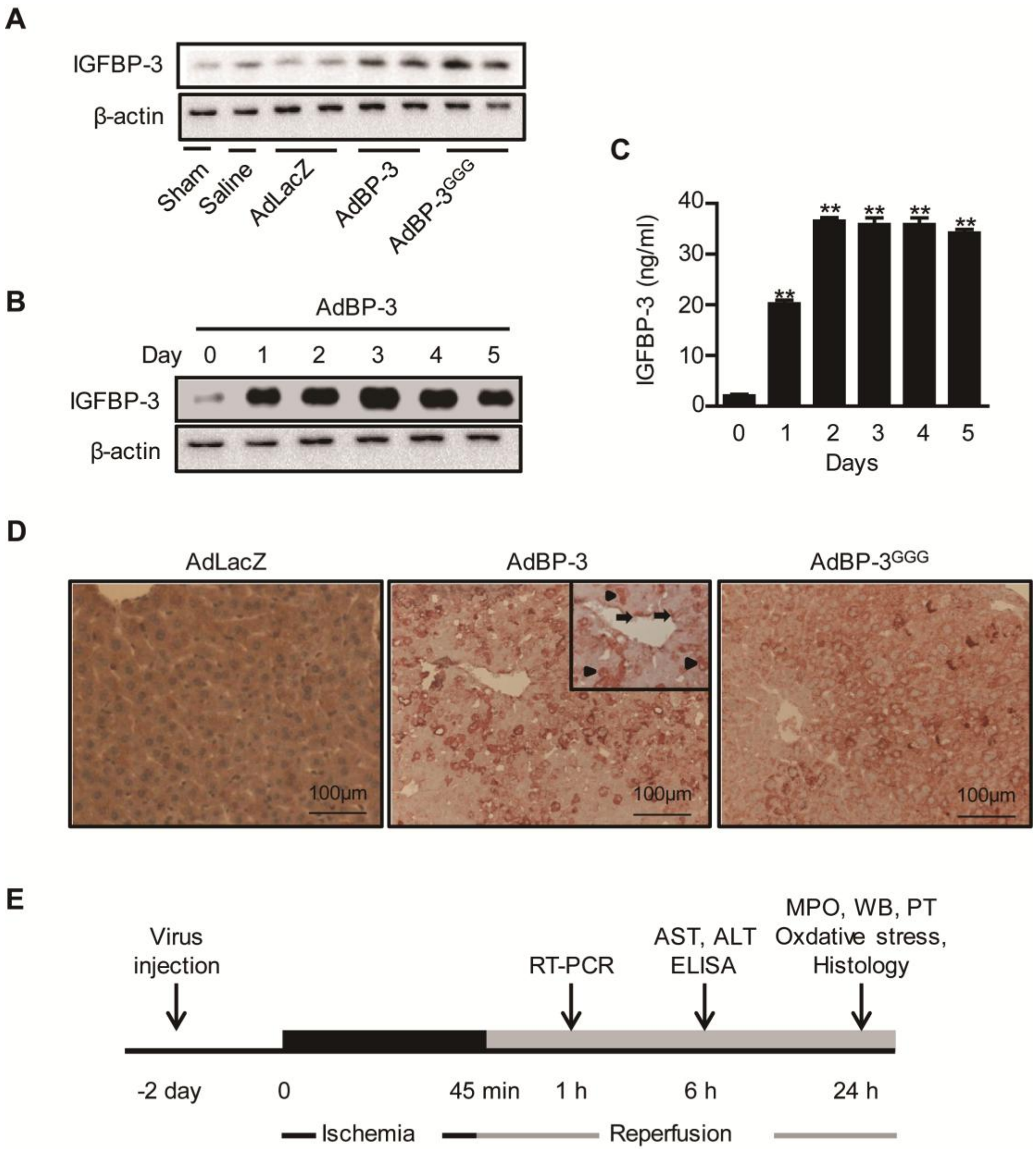

Figure S2

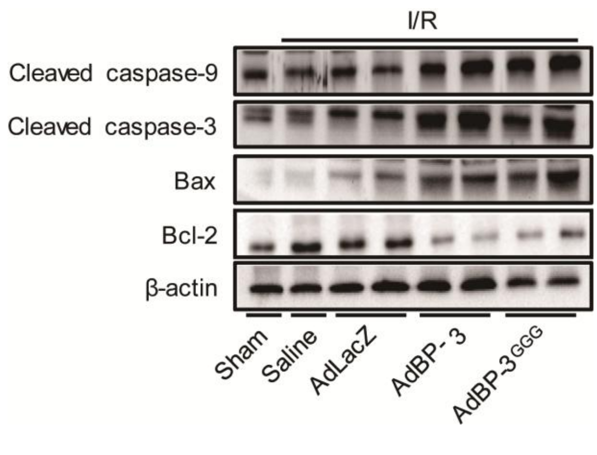
I/R
Cleaved caspase-9
Cleaved caspase-3
Bax
Bcl-2
β-actin
Sham
Saline
AdLacZ
AdBP-3
AdBP-3GGG

Figure S3

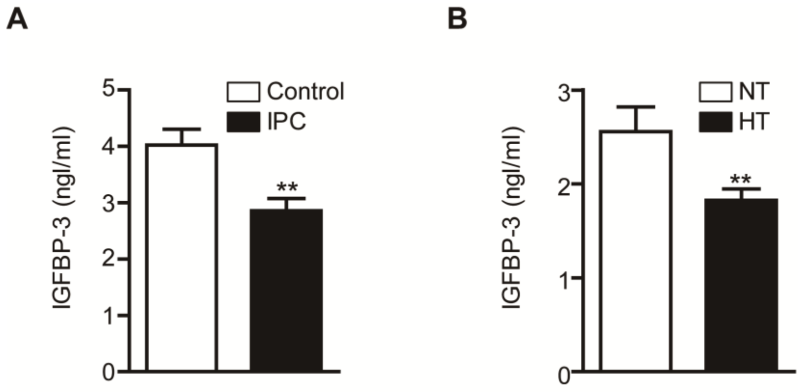

Figure S4

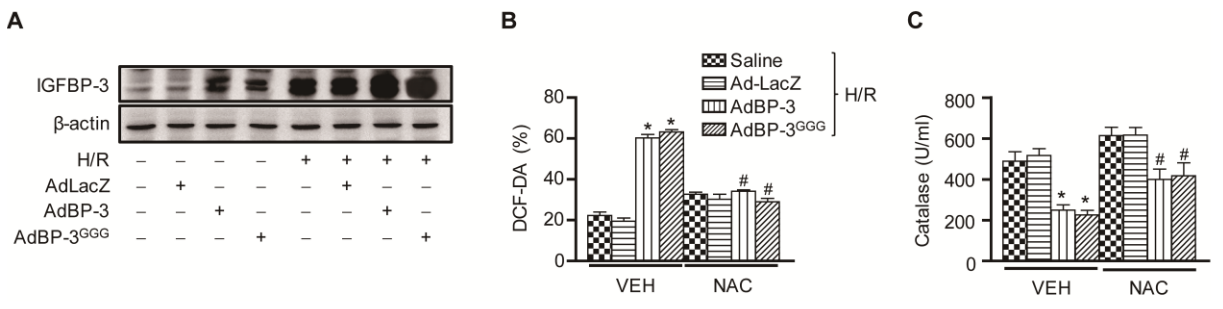
A
IGFBP-3
β-actin
H/R
AdLacZ
AdBP-3
AdBP-3GGG
B
Saline
Ad-LacZ
AdBP-3
AdBP-3GGG
H/R
DCF-DA (%)
VEH
NAC
C
Catalase (U/ml)
VEH
NAC

Figure S5

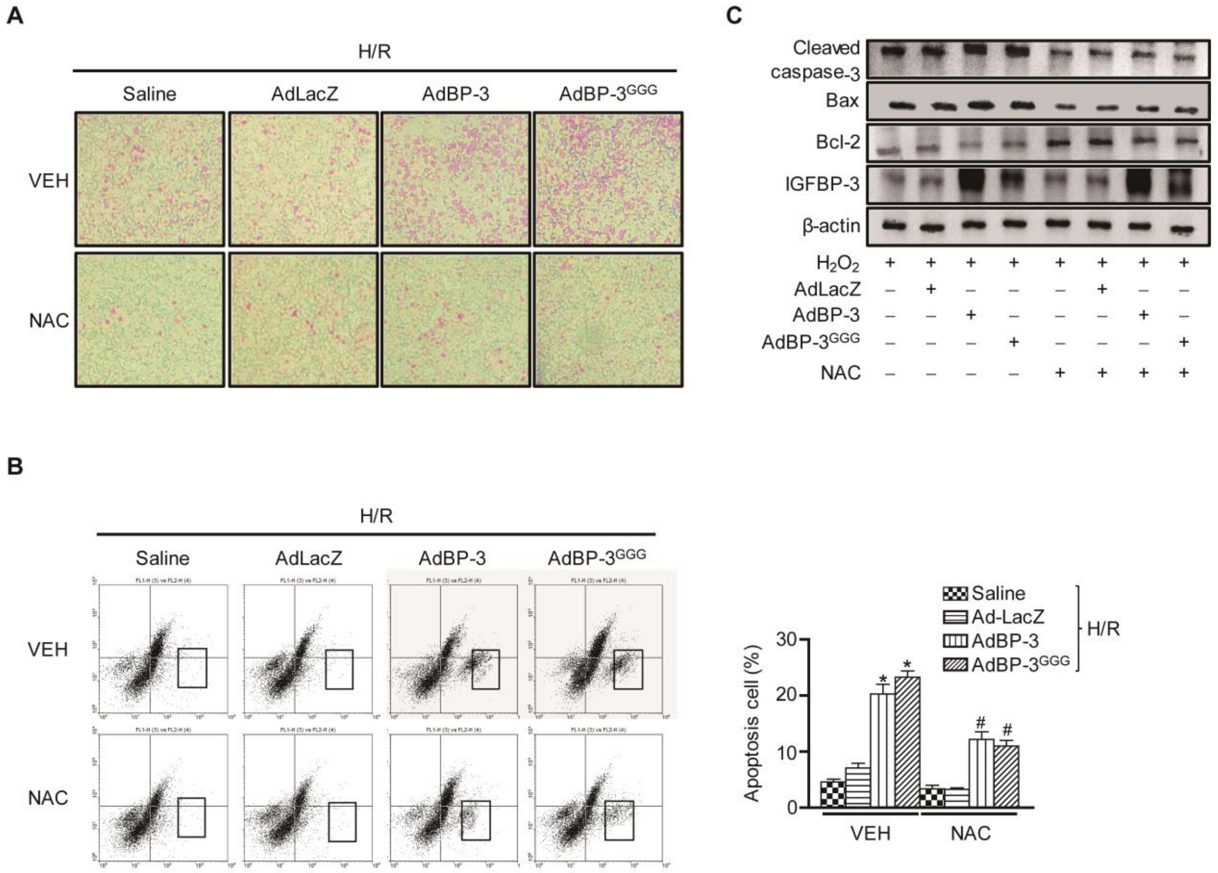
A
H/R
Saline
AdLacZ
AdBP-3
AdBP-3GGG
VEH
NAC
C
Cleaved caspase-3
Bax
Bcl-2
IGFBP-3
β-actin
H2O2
AdLacZ
AdBP-3
AdBP-3GGG
NAC
B
H/R
Saline
AdLacZ
AdBP-3
AdBP-3GGG
VEH
NAC
Saline
Ad-LacZ
AdBP-3
AdBP-3GGG
H/R
Apoptosis cell (%)
30
20
10
0
VEH
NAC
